# Effect of Linearization in a WNT Signaling Model

**DOI:** 10.1155/2019/8461820

**Published:** 2019-06-10

**Authors:** C. F. Ciușdel, S. Coman, Cr. Boldișor, T. Kessler, A. Muradyan, A. Kovachev, H. Lehrach, C. Wierling, L. M. Itu

**Affiliations:** ^1^Department of Automation and Information Technology, R&D Institute of the Transilvania University of Brasov, Brasov 500174, Romania; ^2^Alacris Theranostics GmbH, 12489 Berlin, Germany; ^3^Max Planck Institute for Molecular Genetics, 14195 Berlin, Germany; ^4^Dahlem Centre for Genome Research and Medical Systems Biology, Max-Planck-Str. 3, 12489 Berlin, Germany

## Abstract

A nonlinear model consisting of a system of coupled ordinary differential equations (ODE), describing a biological process linked with cancer development, is linearized using Taylor series and tested against different magnitudes of input perturbations, in order to investigate the extent to which the linearization is accurate. The canonical wingless/integrated (WNT) signaling pathway is considered. The linearization procedure is described, and special considerations for linearization validity are analyzed. The analytical properties of nonlinear and linearized systems are studied, including aspects such as existence of steady state and initial value sensitivity. Linearization is a useful tool for speeding up drug response computations or for providing analytical answers to problems such as required drug concentrations. A Monte Carlo-based error testing workflow is employed to study the errors introduced by the linearization for different input conditions and parameter vectors. The deviations between the nonlinear and the linearized system were found to increase in a polynomial fashion w.r.t. the magnitude of tested perturbations. The linearized system closely followed the original one for perturbations of magnitude within 10% of the base input vector which yielded the state-space fixed point used for the linearization.

## 1. Introduction

The WNT signaling pathway is a cascade of biochemical reactions transducing signals from the extracellular space into the cells. This pathway is involved in different biological processes such as embryonic development, tissue homeostasis, and tumorigenesis [1–3]. The pathway is activated by the binding of WNT-protein ligands to Frizzled family receptors, which pass the biological signal to the Dishevelled (DVL) protein inside the cell.

Specifically, we considered the canonical WNT pathway that regulates translocation of the cytoplasmic *β*-catenin (CTNNB1) into the nucleus. Without WNT signaling, *β*-catenin is degraded by a destruction complex, which includes AXIN, APC, GSK3*β*, and CK. The last two components phosphorylate *β*-catenin on several serine and threonine residues targeting it for ubiquitination and subsequent proteosomal degradation. This continual elimination of the *β*-catenin prevents its translocation to the nucleus [1–3].

The pathway is activated when WNT ligands bind FZD and LRP5/6. This disrupts the function of the destruction complex by recruiting it to the plasma membrane, leading to *β*-catenin stabilization in the cytoplasm and its subsequent nuclear translocation. In the nucleus, *β*-catenin binds TCF/LEF transcription factor proteins and activates expression of WNT target genes [3].

**Figure 1:**
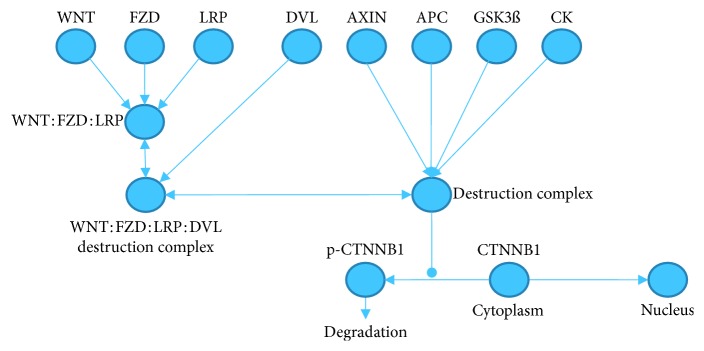
Overview of the WNT signaling model. The WNT signaling pathway model contains 7 WNT ligands, 7 FZD receptors, 2 LRP coreceptors, and 40 readouts.

Nuclear *β*-catenin together with 5 different TCF/LEF transcription factors and 4 other proteins (BCL9, BCL9L, PYGO1, and PYGO2) makes 20 positive readouts. Without WNT signaling and, corresponding nuclear *β*-catenin, the 5 TCF/LEF transcription factors are combined with 4 transcriptional corepressor proteins forming 20 negative readouts, thus leading to a total of 40 readouts.  Figure 1 depicts an overview on the WNT model structure.

The main objective of this paper is to investigate the effect of linearization (based on Taylor series expansion) for a custom prototype model of the human WNT signaling pathway described above, similar to the Reactome model in terms of structure and complexity [4, 5]. The approach considered herein is however generic and applicable to any nonlinear system of coupled ordinary differential equations (ODE).

Linearization may be a useful approach for many signaling model-based use cases. First of all, we refer to the case where only “relative” data are available. “Relative” datasets do not contain pairs of input-output vectors of absolute values. Instead, a data sample represents the ratio of outputs for two different input conditions/vectors. The datasets may be organized in chunks. A chunk has a unique base input vector; other input vectors are variations of the base one. Each input vector has a corresponding output ratio, i.e., the ratio between its corresponding output vector and the base output vector. These ratios may be more relevant than plain absolute values, as they can easily indicate up or down regulation of model components/states for various input conditions/vectors.

To evaluate a ratio, the steady state corresponding to the base input vector must be computed first. Using the base steady-state point as initial conditions, other forward simulations compute the steady state for each input vector. This procedure usually employs numeric integrators for ODE systems, which have various computational and time requirements. A linearization may be conducted around the base steady-state point. For inputs which do not lead to large linearization errors (i.e., ones sufficiently close to the base input vector), the linearized model can be employed since it is faster and easier to use than the nonlinear one.

To compute the numerical solution of stiff nonlinear ODE systems (until steady state is reached) a system of linear equations needs to be solved multiple times. Depending on the time step, and on the number of time steps required to reach the steady state, the total number of solutions may vary between a few tens and several thousands. In contrast, for the linearized system, a system of linear equations must be solved exactly once. Furthermore, additional runtime is required for evaluating the model equations and the Jacobian.

Another advantageous use case for linearization is the computation of the required input deviations (relative to the base input vector) that would lead to a desired change in the state variable vector (relative to the base steady-state point). This approach may be employed to compute the ideal specificity of a potential drug or drug combination to treat a certain type of tumor, or to help in designing new drugs for specific patients or groups of patients.

Moreover, linearization is a step often performed [6–8] in designing control systems for regulating different components of a biological system, either through a nonlinear feedback, or by linearizing the model's equations, as it allows for a direct application of both classical and modern control strategies.

Studying the accuracy of the linearization is therefore useful for indicating when the linearized model can be successfully employed, i.e., when the linearization errors are negligible. These errors depend on the distance between the base input vector and other input vectors. If a suitable threshold can be found, input vectors meeting the threshold can be analyzed using the linearized model, while the rest can be forward simulated using the nonlinear model.

In the following, the base input vector is denoted as *u*_*b*_, and the other input vectors are regarded as input perturbations.

## 2. Methods

### 2.1. Nonlinear Systems and Linearization

Generically, a dynamic nonlinear system is described using the following equations:

(1) $$\begin{array}{c} \frac{dx}{dt}=f\left(x,p,u\right), \end{array}$$

(2) $$\begin{array}{c} y=g\left(x,p,u\right), \end{array}$$

where *x* is the state vector, *p* is the parameter vector, *u* is the input vector, and *y* is the output vector. Function *f* describes the behavior of the system, while function *g* defines the output variables of the model. If *f* is Lipschitz continuous, then equation (1) has a unique solution [9].

For the considered WNT model, *f* and *g* are Lipschitz continuous functions and parameter vector *p* is presumed constant (therefore, its notation can be omitted). The model has the following size: *x* ∈ *ℝ*^421^, *u* ∈ *ℝ*^60^, *p* ∈ *ℝ*^1154^, and *y* ∈ *ℝ*^40^.

If input vector *u* has a step-like evolution, then at a fixed point *x*_ss_ (considered as steady state), equations (1) and (2) are rewritten as follows:

(3) $$\begin{array}{c} 0=f\left(x_{\text{ss}},u_{\text{bf}}\right), \end{array}$$

(4) $$\begin{array}{c} y_{\text{ss}}=g\left(x_{\text{ss}},u_{\text{bf}}\right), \end{array}$$

where *y*_ss_ is the steady-state output vector and *u*_bf_ is the final value of the base input vector *u*_*b*_.

If a linearization is performed around *x*_ss_, using Taylor series expansion, and all terms of second or higher order are being discarded, then

(5) $$\begin{array}{c} f_{\text{linear}}\left(x,u\right)=f\left(x_{\text{ss}},u_{\text{bf}}\right)+\frac{\partial f}{\partial x}\left(x_{\text{ss}},u_{\text{bf}}\right)\left(x-x_{\text{ss}}\right)+\frac{\partial f}{\partial u}\left(x_{\text{ss}},u_{\text{bf}}\right)\left(u-u_{\text{bf}}\right), \end{array}$$

where (∂*f*/∂*x*)*x*_ss_, *u*_bf_ is the state Jacobian evaluated at (*x*_ss_, *u*_bf_) and (∂*f*/∂*u*)*x*_ss_, *u*_bf_ is the input Jacobian evaluated at (*x*_ss_, *u*_bf_). Vectors (*x* − *x*_ss_) and (*u* − *u*_bf_) are states and input vector deviations from the point where the linearization was performed at.

Typically, the following notations are used in control theory [10, 11]:

(6) $$\begin{array}{c} A=\frac{\partial f}{\partial x}\left(x_{\text{ss}},u_{\text{bf}}\right), \end{array}$$

(7) $$\begin{array}{c} B=\frac{\partial f}{\partial u}\left(x_{\text{ss}},u_{\text{bf}}\right). \end{array}$$

If conditions (i-ii) listed below hold, the nonlinear system is already linear in its input space and the linearization does not introduce any errors from omitting nonlinear terms of (*u* − *u*_bf_) since they do not exist. These conditions are true for the WNT model considered in this paper:

- The input Jacobian is constant (i.e., does not depend on *x* or *u*)
- The state Jacobian does not depend explicitly on *u*

Using equations (3) and (6)-(7),

(8) $$\begin{array}{c} \frac{dx_{\text{linear}}}{dt}=f_{\text{linear}}\left(x_{\text{linear}},u\right)=A\left(x_{\text{linear}}-x_{\text{ss}}\right)+B\left(u-u_{\text{bf}}\right), \end{array}$$

where *x*_linear_ is the state vector of the linearized system.

Based on equation (3), at the point of linearization, *dx*_ss_/*dt* is zero. Therefore,

(9) $$\begin{array}{c} \frac{d}{dt}\left(x_{\text{linear}}-x_{\text{ss}}\right)=A\left(x_{\text{linear}}-x_{\text{ss}}\right)+B\left(u-u_{\text{bf}}\right). \end{array}$$

If *x*_dev_ is defined as (*x*_linear_ − *x*_ss_), i.e., the vector representing the deviations of the state from the point of linearization for the linear model, then

(10) $$\begin{array}{c} \frac{dx_{\text{dev}}}{dt}=Ax_{\text{dev}}+B\left(u-u_{\text{bf}}\right). \end{array}$$

The agreement between the linearized model and the original model can be formalized as follows:

(11) $$\begin{array}{c} \frac{dx_{\text{dev}}}{dt}=Ax_{\text{dev}}+B\left(u-u_{\text{bf}}\right),\\ \frac{dx_{\text{nonlin}}}{dt}=f\left(x_{\text{nonlin}},u\right),\\ e=x_{\text{nonlin}}-x_{\text{dev}}-x_{\text{ss}}, \end{array}$$

where *dx*_nonlin_/*dt* is the state evolution as dictated by the original nonlinear system and *e* is the error vector representing the differences introduced by the linearization. A new nonlinear system was obtained, describing the evolution in time of the agreement between the linearized and the original model. By defining

(12) $$\begin{array}{c} x_{\text{agreement}}=\left[\begin{array}{c} x_{\text{dev}}\\ x_{\text{nonlin}} \end{array}\right], \end{array}$$

an analysis related to the errors introduced by the linearization can be conducted for different input vectors *u*. An example workflow would be as follows:

- Forward simulate the nonlinear model until a steady-state point *x*_ss_ is reached for input *u*_*b*_
- Compute matrices *A* and *B*
- Forward simulate model (11) for a new input vector *u*, with the initial conditions $x_{{\text{agreement}}_{\text{0}}}=\left[\begin{array}{c} 0\\ x_{\text{ss}} \end{array}\right]$
- Compute measures of interest on the agreement error vector *e*

### 2.2. Effects of Linearization

If the linearization is performed around a hyperbolic fixed point, the Hartman–Grobman theorem [12] guarantees that the linearized system will exhibit the same qualitative behavior as the original system, in a sufficiently small neighborhood around the linearization point. A fixed point *x*_*f*_ is “hyperbolic” if all eigenvalues of the Jacobian evaluated at *x*_*f*_ (i.e., the linearized systems' poles) have a nonzero real part (i.e., the system does not have purely oscillatory modes).

In terms of quantitative behavior, usually a linearization is considered accurate only for small deviations of the state vector around an equilibrium point. Of importance is the steady-state behavior of the linearized system when compared to the original one, i.e., the steady-state deviations/errors (introduced by the simplified model) for different magnitudes of exogenous input perturbations.

In case of stable linearized models, for step-like inputs, the steady state is dependent only on the final value of the exogenous input vector and on the model matrices:

(13) $$\begin{array}{c} 0=\frac{dx_{{\text{dev}}_{\text{ss}}}}{dt}=Ax_{{\text{dev}}_{\text{ss}}}+B\left(u_{t⟶\infty }-u_{\text{bf}}\right),\\ x_{{\text{dev}}_{\text{ss}}}=-A^{-1}B\left(u_{t⟶\infty }-u_{\text{bf}}\right). \end{array}$$

Herein, all vector fields *u* which have the following properties are regarded as “step-like” inputs:

(14) $$\begin{array}{c} u_{t⟶\infty }=\underset{t⟶\infty }{\lim}u\left(t\right)=u_{\text{final}}=\text{const},\\ u_{\text{final}}\neq u_{\text{bf}}. \end{array}$$

This translates to the new steady state depending neither on the time-dependent evolution of the input vector (it depends only on its final value) nor on the initial conditions *x*_0_.

However, these considerations are not universally true for nonlinear systems. The existence of the steady state is guaranteed if the following lemma applies. Suppose *B* is a nonempty bounded subset of *ℝ*^*n*^. By definition [13], the *ω* limit of set *B*, denoted *ω*(*B*), is the set of all points *x* for which a sequence of pair {*x*_*k*_, *t*_*k*_} exists, with *x*_*k*_ ∈ *B* and $\underset{k⟶\infty }{\lim}t_{k}=\infty$ such that

(15) $$\begin{array}{c} \underset{k⟶\infty }{\lim}x\left(t_{k},x_{k}\right)=x. \end{array}$$

**Lemma 1 .**

[13]. Let *B* be a nonempty bounded subset of *ℝ*^*n*^ and suppose there is a number *M* such that *x*(*t*, *x*_0_) ≤ *M* for all *t* ≥ 0 and all *x*_0_ ∈ *B*. Then, *ω*(*B*) is a nonempty compact invariant set. Moreover, the distance of *x*(*t*, *x*_0_) from *ω*(*B*) approaches 0 as *t*⟶*∞*, uniformly in *x*_0_ ∈ *B*.

Less formally stated, if the nonlinear system has an overall stable behavior, i.e., with initial conditions *x*_0_ in a neighborhood *B*, its state does not diverge towards infinity, then at a large enough time *t* ≥ *T*, its state trajectory will enter and remain in a closed set *ω*(*B*). If neighborhood *B* is centered around an attractor fixed point *x*_ss_, which can be used as a linearization point, the lemma suggests, if its conditions are met, that both the linearized and the nonlinear system will reach a new steady state in case of an exogenous perturbation. It is worth noting that the lemma deals explicitly with autonomous systems, but, if the evolution of the exogenous perturbation is known, it can be integrated into an extended autonomous system, comprising two subsystems: the perturbation model and the original nonlinear model [14]. In the following, let *ω*(*B*) consist of only one point, i.e., the system has no oscillatory behavior at steady state.

To analyze the influence of the initial condition *x*_0_ on the state trajectory of the nonlinear model, the Lyapunov exponents theory can be employed. We consider Φ as the discrete-time counterpart of the continuous-time model *f*, with the property that *x*_*k*+1_=Φ(*x*_*k*_), where *x*_*k*_=*x*(*t*_0_+*kT*_*s*_) and *T*_*s*_ is the sampling period. The following statement holds (any conclusions drawn for the discrete model are also valid for the continuous one): the global Lyapunov exponent of Φ at *x* with respect to direction *y* is defined as the limit (if it exists) [15]:

(16) $$\begin{array}{c} \underset{t⟶\infty }{\lim}\frac{1}{t}\underset{\delta ⟶0}{\lim}\ln\frac{\left\|\Phi^{t}\left(x+\delta y\right)-\Phi^{t}\left(x\right)\right\|}{\left\|\delta y\right\|}=\lambda , \end{array}$$

where Φ^*k*^(*x*_0_)=*x*(*t*_*k*_, *x*_0_). The global Lyapunov exponent *λ* describes the decay in time of the distance between two trajectories which start at two separate initial points *x* and *x*+*δy*:

(17) $$\begin{array}{c} \frac{\left\|\Phi^{t}\left(x+\delta y\right)-\Phi^{t}\left(x\right)\right\|}{\left\|\delta y\right\|}\approx e^{\lambda t}. \end{array}$$

If *λ* < 0, any state trajectory which starts at *x*+*δy* (for any sufficiently small *δ*) will converge to the “main” trajectory which starts at *x*. Since for a small *δ* we can state that

(18) $$\begin{array}{c} \left\|\Phi \left(x+\delta y\right)-\Phi \left(x\right)\right\|\approx \left\|J\left(\Phi ,x\right)\delta y\right\|, \end{array}$$

where *J*(Φ, *x*) is the Jacobian of Φ evaluated at *x* and *λ* can be expressed as

(19) $$\begin{array}{c} \lambda =\underset{n⟶\infty }{\lim}\frac{1}{n}\ln\frac{\left\|J\left(\Phi^{n},x\right)y\right\|}{\left\|y\right\|}=\frac{1}{n}\overset{n-1}{\underset{t=0}{\sum }}\ln\frac{\left\|J\left(\Phi^{t+1},x\right)y\right\|}{\left\|J\left(\Phi^{t},x\right)y\right\|}=\frac{1}{n}\overset{n-1}{\underset{t=0}{\sum }}\ln\frac{\left\|J\left(\Phi ,\Phi^{t}\right)J\left(\Phi^{t},x\right)y\right\|}{\left\|J\left(\Phi^{t},x\right)y\right\|}. \end{array}$$

Thus, the global Lyapunov exponent of Φ at point *x* with respect to direction *y* is the average of the local Lyapunov exponents of Φ at points *x*_0,_*x*_1_, *x*_2_,… of the trajectory of *x* w.r.t direction *J*(Φ^*k*^, *x*)*y* at each point *x*_*k*_ [15]. The local Lyapunov exponents are characterized by the eigenvalues of *J*(Φ, *x*_*k*_). Therefore, if the system reaches a new steady state (e.g., an attractor fixed point *x*_ssfinal_) when an input perturbation is applied (starting from an initial stable equilibrium point *x*_ss0_ which can be employed for the linearization), all state trajectories starting from a sufficiently small neighborhood *B* of *x*_ss0_ will converge to *x*_ssfinal_.

Let *S* ⊆ *ℝ*^*n*^ be a compact and convex set of points *x* for which the state Jacobian evaluated at *x* has negative real-part eigenvalues. More formally, let *λ*_*i*_ be the eigenvalues of (∂*f*/∂*x*)*x*, *p*, and *ℜ*(*λ*_*i*_) < 0, ∀*x* ∈ *S*, *i* ∈ [1,…, *n*]. In this case, the eigenvalues of the discrete-time model Φ lie inside the unit circle. *S* is restricted so that Φ(*x*) ∈ *S*, ∀*x* ∈ *S*. Then, at each point *x* ∈ *S*, the local Lyapunov exponent w.r.t. direction *y* is defined as

(20) $$\begin{array}{c} \underset{\delta ⟶0}{\lim}\ln\frac{\left\|\Phi \left(x+\delta y\right)-\Phi \left(x\right)\right\|}{\left\|\delta y\right\|}, \end{array}$$

and is negative; therefore,

(21) $$\begin{array}{c} \left\|\Phi \left(x+\Delta y\right)-\Phi \left(x\right)\right\|\leq c\left\|\Delta y\right\|, \end{array}$$

where 0 < *c* < 1 and Δ > 0 is a small enough number.

It can be shown that, for a constant input *u*, for every initial point *x*_0_ ∈ *S*, the nonlinear model reaches a unique fixed point *x*_ss_ ∈ *S* at steady state. Banach's fixed point theorem states that if Φ is a strict contraction, then Φ has a unique fixed point in *S* [9]. A map Φ is a strict contraction on *S*, if ∀*p*, *q* ∈ *S*:

(22) $$\begin{array}{c} \left\|\Phi \left(p\right)-\Phi \left(q\right)\right\|\leq c\left\|p-q\right\|, \end{array}$$

where *c* < 1. To prove equation (22), consider the line segment connecting points *p* and *q*, and the sequence of distinct points *p*_1_, *p*_2_,…*p*_*k*_, *q*_*k*_, *q*_*k*−1_,…, *q*_1_ between *p* and *q*. Then,

(23) $$\begin{array}{c} \left\|\Phi \left(p\right)-\Phi \left(q\right)\right\|=\left\|\Phi \left(p\right)-\Phi \left(p_{1}\right)-\left(\Phi \left(q\right)-\Phi \left(q_{1}\right)\right)+\Phi \left(p_{1}\right)-\Phi \left(q_{1}\right)\right\|\leq \\ \leq \;\;\left\|\Phi \left(p\right)-\Phi \left(p_{1}\right)\right\|+\left\|\Phi \left(q\right)-\Phi \left(q_{1}\right)\right\|+\left\|\Phi \left(p_{1}\right)-\Phi \left(q_{1}\right)\right\|. \end{array}$$

The points of each pair (*p*_*k*−1_, *p*_*k*_) are close enough:

(24) $$\begin{array}{c} \left\|\Phi \left(p_{k-1}\right)-\Phi \left(p_{k}\right)\right\|\leq c_{p_{k}}\left\|p_{k-1}-p_{k}\right\|, \end{array}$$

where *c*_*p*_*k*__ < 1 is the local Lyapunov exponent of Φ at *p*_*k*_. Applying the procedure of equation (23) iteratively and employing equation (24) yields

(25) $$\begin{array}{c} \left|\left|\Phi \left(p\right)-\Phi \left(q\right)\right|\right|\leq c_{p_{1}}\left\|p-p_{1}\right\|+\ldots +c_{p_{k-1}}\left\|p_{k}-p_{k-1}\right\|+\ldots +c_{q_{1}}\left\|q-q_{1}\right\|. \end{array}$$

Let *c*_max_=max{*c*_*p*_1__,…, *c*_*p*_*k*−1__,…, *c*_*q*_1__} < 1.

Then,

(26) $$\begin{array}{c} \left\|\Phi \left(p\right)-\Phi \left(q\right)\right\|\leq c_{\max}\left(\left\|p-p_{1}\right\|+\ldots +\left\|p_{k}-p_{k-1}\right\|+\ldots +\left\|q-q_{1}\right\|\right). \end{array}$$

Because *p*, *p*_1_, *p*_2_,…*p*_*k*_, *q*_*k*_, *q*_*k*−1_,…, *q*_1_, *q* are collinear points, their inner distances sum to ‖*p* − *q*‖. Therefore,

(27) $$\begin{array}{c} \left\|\Phi \left(p\right)-\Phi \left(q\right)\right\|\leq c_{\max}\left\|p-q\right\|, \end{array}$$

and Φ is a strict contraction for all *p*, *q* ∈ *S*. This also implies that the *ω* limit of *S* consists of only one point. For comparison, a stable linear system will always reach a unique steady-state fixed point (i.e., ∀*x*_0_ ∈ *ℝ*^*n*^ at *t*_0_) for a specific constant input, as stated in equation (13).

As the global Lyapunov exponent is the average of the local Lyapunov exponents, the existence of *S* is a particular case of the global Lyapunov exponent reasoning described above. *S* may also be regarded as a smaller subset of the attraction basin of the attractor fixed point *x*_ss_.

In conclusion, a linearization offers valid results only when tested against valid input perturbations, i.e., perturbations that do not drive the nonlinear system into an unstable region. In this case, the nonlinear and linearized models have similar behaviors: existence of steady state and (local) insensitivity w.r.t. initial conditions. The application of an exogenous input perturbation modifies the location in the state space of the attractor fixed point *x*_*ss*_, and therefore, it invalidates the prerequisites of the Hartman–Grobman theorem (due to the fact that the linearized system was defined at the initial fixed point location *x*_ss0_, before applying the exogenous perturbation). Although the trajectories of the models will initially behave qualitatively the same when starting from *x*_ss0_, i.e., the trajectories will start varying in the same direction (since the linearization is accurate for small deviations around *x*_ss0_), the two models may behave qualitatively differently around *x*_ss_. This holds true especially if the Jacobian varies considerably between *x*_ss_ and *x*_ss0_.

The actual steady-state error between the linearized model and the original model is influenced by the Hessian of the system. For exemplification, suppose conditions (i-ii) hold, let *x*_lin_ be an attractor fixed point around which a linearization was performed, and the input perturbation *u* be a Heaviside step function. As a first example, consider the Taylor expansion of *f* around *x*_lin_:

(28) $$\begin{array}{c} f\left(x,u\right)=f\left(x_{\text{lin}},u_{\text{bf}}\right)+\frac{\partial f\left(x_{\text{lin}}\right)}{\partial x}\left(x-x_{\text{lin}}\right)+\frac{1}{2!}{\left(x-x_{\text{lin}}\right)}^{\text{T}}\frac{\partial^{2}f\left(x_{\text{lin}}\right)}{\partial x^{2}}\left(x-x_{\text{lin}}\right)+\ldots +B\left(u-u_{\text{bf}}\right). \end{array}$$

At a new steady state of the nonlinear model *x*_ss_, *f*(*x*_ss_, *u*) will be zero. If the Hessian norm is relatively large, the large weights of the second-order term will introduce errors between the steady state of the linearized and the steady state of the original model.

As a second example, computing the partial derivatives of *x* w.r.t. *u* at both fixed points *x*_lin_ and *x*_ss_ yields

(29) $$\begin{array}{c} \frac{\partial x_{\text{lin}}}{\partial u}=-{\left(\frac{\partial f\left(x_{\text{lin}},u\right)}{\partial x}\right)}^{-1}B=-A^{-1}B,\\ \frac{\partial x_{\text{ss}}}{\partial u}=-{\left(\frac{\partial f\left(x_{\text{ss}},u\right)}{\partial x}\right)}^{-1}B. \end{array}$$

If the Jacobian varies significantly between the two fixed points *x*_lin_ and *x*_ss_, the linearized and the original system will exhibit different input sensitivities. The rate of change of the Jacobian w.r.t. *δx* ≈ *x*_ss_ − *x*_lin_ is dictated by the Hessian.

As a third example, by following an iterative procedure, one can forward simulate the linearized system for small enough time increments so that it follows precisely the nonlinear system. More formally, there is a *δ* > 0 so that, for every *ε* > 0, if ‖*x*_nonlinear_(*t*) − *x*_linear_(*t*) < *ε*‖, then Δ*t*=*t*_*k*+1_ − *t*_*k*_ < *δ*. At the end of each time increment, the linearization (i.e., the Jacobian) is recomputed. Applying this for *k* steps until the new steady state is reached yields

(30) $$\begin{array}{c} 0=f\left(x_{\text{ss}},u\right)=\underset{k}{\sum }\frac{\partial f\left(x_{k}\right)}{\partial x}\left(x_{k+1}-x_{k}\right)+B\left(u-u_{\text{bf}}\right). \end{array}$$

If the Jacobian is constant, equation (30) reduces to equation (13). Therefore, if the Jacobian varies significantly along the state trajectory towards the new attractor point given by the applied perturbation, the linearized system will deviate from the real one. Again, if the Hessian computed at *x*_lin_ has a relatively large norm, linearization errors are to be expected.

### 2.3. Properties of the Considered WNT Model

By analyzing the model equations, we observe that function *f* has for all state variables the following form:

(31) $$\begin{array}{c} {\dot{x}}_{k}=f_{k}\left(x,p,u\right)=-x_{k}h_{1_{k}}\left(x_{i},p\right)+h_{2_{k}}\left(x_{i},p\right)+u_{k}, \end{array}$$

where *x*_*k*_ is the *k*^th^ state variable, *x*_*i*_ is the set of all state variables excluding *x*_*k*_ (i.e., *i* ∈ [1,…, *k* − 1, *k*+1,…, 421]), and *h*_1_*k*__ and *h*_2_*k*__ are nonlinear Lipschitz continuous functions with the property that *h*_1_*k*__(*x*, *p*) > *c*_*k*_ > 0 and *h*_2_*k*__(*x*, *p*) ≥ 0, ∀*x*, *p* > 0, and *c*_*k*_ is constant. When both the exogenous input *u*_*k*_ and the parameter vector *p* are chosen to be positive (which is the case for the considered datasets) and all state variables are nonnegative, the subsystem defined by *f*_*k*_ is equivalent to a stable linear time-variant first-order filter. This, however, does not imply that the entire model is stable, due to the existence of nonlinear feedback loops.

If all state variables have nonnegative initial values (at *t*_0_), *x*_*k*_ will always remain nonnegative. To prove this, let all other state variables be nonnegative and consider the zerocrossing of *x*_*k*_. Equation (31) is reduced to

(32) $$\begin{array}{c} {\dot{x}}_{k}=f_{k}\left(x,p,u\right)=h_{2_{k}}\left(x_{i},p\right)+u_{k}\geq 0. \end{array}$$

Therefore, *x*_*k*_ will increase again as a positive number. As a consequence, negative values have no relevance for the nonlinear model and can simply be corrected in the linearized model. The predictions of the linearized model were thus in the end adjusted by clipping the state variable values in the range [0, *∞*).

### 2.4. Monte Carlo Analysis of Linearization Accuracy Range

To estimate the quantitative behavior of the linearized model, the following workflow was used:

1. Generate a random parameter vector *p* and set *r*_noise_, representing the range of a uniform distribution centered around 1, used to perturb the input vectors
2. For each input sample *u*_*b*,*i*_,
  - Forward simulate the nonlinear system until the steady-state *x*_ss0_ is reached
  - Linearize the nonlinear model around *x*_ss0_
  - Compute *u*_*i*_ by perturbing input vector *u*_bf,*i*_, with a multiplicative noise of range ±*r*_noise_%
  - Forward simulate system (11) until the steady state, using the initial conditions $x_{\text{agreement0}}=\left[\begin{array}{c} 0\\ x_{\text{ss0}} \end{array}\right]$
3. Compute metrics by averaging over all input samples, for the current *p* and *r*_noise_.

## 3. Results

For the WNT model described Introduction, the proposed workflow was applied four times, for different parameter vectors *p*. Three ranges *r*_noise_ of multiplicative noise were used: 10%, 25%, and 50%. Vectors *p* were drawn from a log-uniform distribution over [10^−1^, 10^1^]. The same set of inputs *u*_bf,*i*_ was used for all workflow runs, and it consisted of 2000 synthetically generated input samples, drawn from a fitted log-normal distribution over a smaller set of real input samples. The perturbed input was computed as

(33) $$\begin{array}{c} u_{i}=\left(1+r_{\text{noise}}-2r_{\text{noise}}d_{i}\right)\;\circ \;u_{\text{bf},i}, \end{array}$$

where “∘” is the Hadamard product and *d*_*i*_ is the perturbation direction for input *u*_*i*_. Vectors *d*_*i*_ were drawn from a uniform distribution over [0, 1]. Each workflow run had its own set of directions *d*_*i*_, used for all ranges *r*_noise_.

First of all, we refer to runtime considerations: a runtime larger than 0.150 seconds is required for a nonlinear simulation to steady state of the herein considered WNT model (based on Matlab's ODE15 s function, with user-supplied Jacobian), whereas for the linearized system, only 0.003 seconds are required. This time difference may become significant, especially when the “relative” dataset chunk size is considerable.

Tables 1 and 2 display the steady-state results (averaged over all input samples) for the four runs of the proposed workflow. The predictions output by the linearized model were corrected as described above, to avoid negative-state variable values. For Table 1, the results were averaged over all model states, while for Table 2, the results were averaged over the model outputs (i.e., the readout model states). MRE and MSE represent mean relative and mean squared errors, respectively:

(34) $$\begin{array}{c} \text{MSE}=\bar{{\left(x_{\text{ss linear}}-x_{\text{ss nonlinear}}\right)}^{2}}, \end{array}$$

(35) $$\begin{array}{c} \text{MRE}=\bar{\left|\frac{x_{\text{ss linear}}-x_{\text{ss nonlinear}}}{x_{\text{ss nonlinear}}}\right|}, \end{array}$$

where *x*_ss linear_ and *x*_ss nonlinear_ are the linear and nonlinear steady-state vectors (after having applied perturbation) and std(*e*)/std(*x*_diff_) is the ratio between the standard deviation of the linearization error vector and the standard deviation of the vector *x*_diff_=*x*_ss nonlinear_ − *x*_ss0_, where *x*_ss0_ is the linearization point.

**Figure 2:**
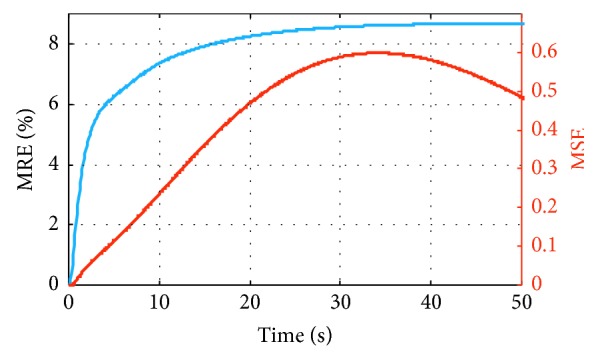
Evolution in time of the error metrics for one perturbation example with *r*_noise_=50%.

As an example, we display in Figure 2 the evolution in time of the error metrics MRE and MSE for a single case with *r*_noise_=50%. The errors increase in time, as the state vector moves farther away from the linearization point *x*_ss0_, and consequently, the actual Jacobian differs more substantially from the one computed at *x*_ss0_.

**Figure 3:**
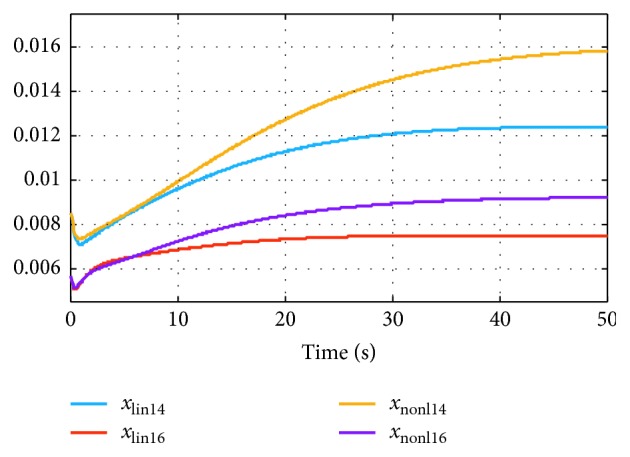
Evolution in time of two state variables, for both systems, with *r*_noise_=50%.

In Figure 3, we display the evolution in time for two model states for which the linearization introduces errors (the same case as in Figure 2 was considered). At first, the two models display almost perfect agreement (both qualitatively and quantitatively). However, as the state vector starts departing farther away from the linearization point (at *t*=0), the error between the two models increases.

**Figure 4:**
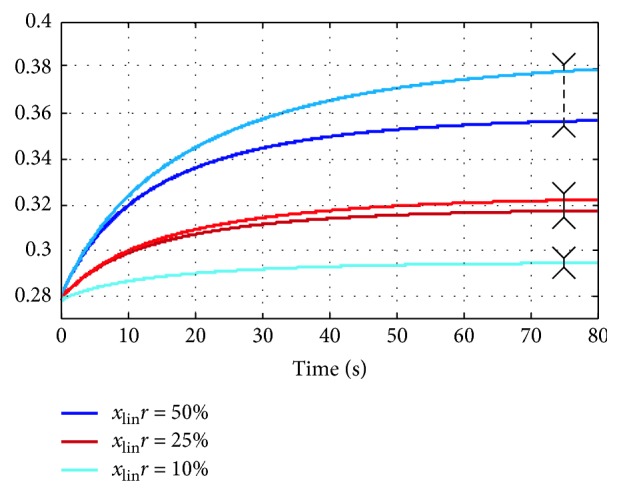
Evolution in time of one state variable for the linear and the nonlinear systems, for all three perturbation levels. The differences between the two models are indicated with arrows. The linear model is depicted with darker colors. For *r*_noise_=10%, the two models overlap perfectly.

**Figure 5:**
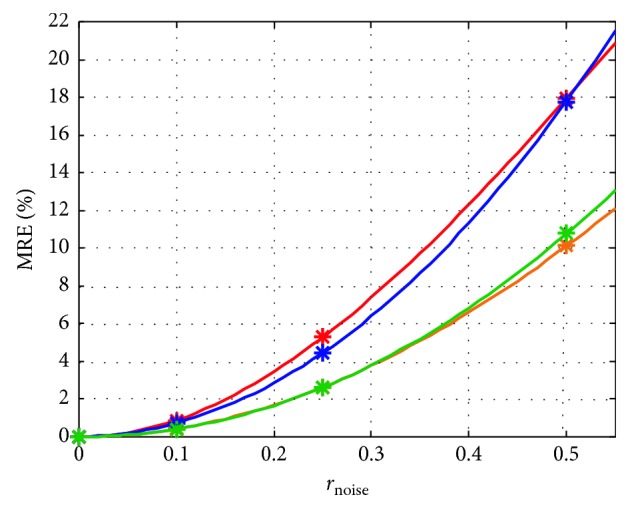
Dependence between perturbation level *r*_noise_ and the mean relative linearization error of the model outputs, at steady state and for the four workflow runs.

Figure 4 displays the dependence between linearization error and *r*_noise_ for one state variable of the model. The evolution in time of both models, linearized and nonlinear, is depicted, for all three values of *r*_noise_. For small perturbations, the linearized model follows closely the nonlinear version. As the perturbations become larger in magnitude, the difference between the time courses of the two models increases. This behavior can be observed also in Figure 5, where the MRE variation (for the output variables of the model, in steady state) was plotted for all workflow runs. An approx. polynomial dependence can be observed, which is in agreement with equation (28).

**Figure 6:**
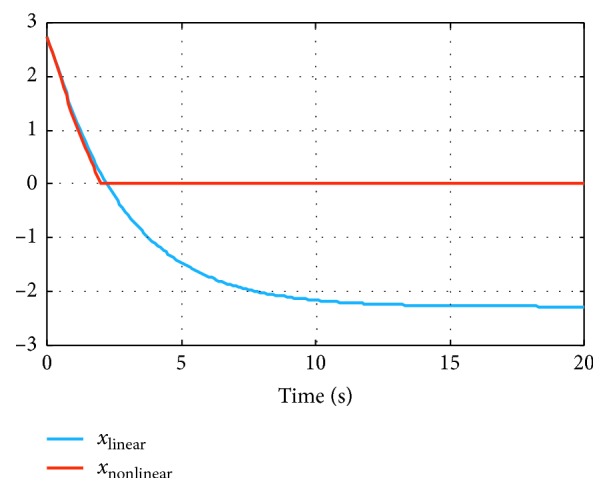
An example which yields negative values for one of the linearized model state variable. The nonlinear state variable decays to zero, while the state variable in the linearized model converges to a negative value.

Without the adjustment introduced for the predictions of the linearized model, for some input perturbations, certain state variables in the nonlinear model decay to zero, while the state variables in the linearized model converge to negative values. An example is displayed in Figure 6. The two models display a good agreement up to the zerocrossing of the state variable in the linearized model. Without the adjustment, this example would have yielded a high MRE value since the linearization error would have been divided by a small denominator, according to equation (35).

## 4. Conclusions

Linearization is a useful tool for the analysis of large nonlinear dynamical systems. It produces a simpler version of the original model, on which established linear analysis methods can be applied to gain insights into the local behavior of the original model.

As an example to test the approach, a model of the canonical WNT signaling pathway was considered. The model was forward simulated until steady state was reached for an input set *u*_*b*,*i*_. Linearization was performed at steady state for each input sample, and the accuracy of the linearized model was determined for various ranges of input perturbation magnitudes.

The concepts related to linearization validity were presented. A linearization approach offers correct insights only when tested against valid input perturbations (i.e., ones that do not drive the nonlinear system into an unstable region).

In terms of accuracy, errors tend to increase in a polynomial fashion w.r.t. the magnitude of tested perturbations. For small enough perturbations, the linearized system closely follows the original one.

By choosing an acceptable error level, a relative input perturbation threshold can be derived, which can be used to discriminate between various test input vectors, i.e., predict which input vectors will not lead to large linearization errors. For the considered WNT model, linearization errors were determined to be low for input vectors *u*_*i*_ within ±10% of the base input vector *u*_bf,*i*_.

## Figures and Tables

**Table 1:** Metrics on all states, computed using adjusted linear predictions.

Workflow number		1	2	3	4
*r*_noise_=±10%	std(*e*)/std(*x*_diff_) (%)	4.61	4.89	5.08	5.53
	MRE (%)	0.41	0.27	0.59	0.31
	MSE	0.162	0.024	0.195	0.039

*r*_noise_=±25%	std(*e*)/std(*x*_diff_) (%)	10.41	12.09	11.60	12.90
	MRE (%)	2.33	1.65	3.38	1.86
	MSE	4.763	0.895	6.217	1.044

*r*_noise_=±50%	std(*e*)/std(*x*_diff_) (%)	18.67	22.68	20.43	22.92
	MRE (%)	9.10	6.13	12.29	6.48
	MSE	62.042	13.702	85.323	17.656

**Table 2:** Metrics on model outputs, computed using adjusted linear predictions.

Workflow number		1	2	3	4
*r*_noise_=±10%	std(*e*)/std(*x*_diff_) (%)	9.65	7.05	6.95	7.33
	MRE (%)	0.87	0.40	0.72	0.39
	MSE	6.80E−04	1.08E−04	4.50E−05	3.30E−05

*r*_noise_=±25%	std(*e*)/std(*x*_diff_) (%)	22.51	17.53	16.76	17.34
	MRE (%)	5.26	2.62	4.43	2.61
	MSE	2.70E−02	4.52E−03	1.32E−03	1.28E−03

*r*_noise_=±50%	std(*e*)/std(*x*_diff_) (%)	43.12	31.61	30.73	31.81
	MRE (%)	17.96	10.12	17.80	10.76
	MSE	3.89E−01	6.06E−02	2.76E−02	1.95E−02

## Data Availability

The data used to support the findings of this study are available from the corresponding author upon request.
